# Supplementary material for: A Likelihood Approach for Real-Time Calibration of Stochastic Compartmental Epidemic Models
Source: PLoS Comput Biol. 2017 Jan 17;13(1):e1005257. doi: 10.1371/journal.pcbi.1005257 (PMC5240920; doi:10.1371/journal.pcbi.1005257)
Supplement: S1 File — (TAR.GZ) [file pcbi.1005257.s014.tar.gz › HSPH_Online-SI-Revision/Additions/MSS11/n100-extreme_noisy/n100-extreme_noisy_NEW2_table-8.pdf]

1	newly 3 weeks spec	1833.	3657.07	{1205., 5680.}	114.262	0.371386	newly 3 weeks cumul	3045.73	6076.85	{2052., 9472.}	113.55	0.355432	newly 5 weeks spec	5869.58	10 003.4	{4029., 13 931.}	84.3506	0.325236	newly 5 weeks cumul	12 428.	22 821.7	{9110., 33 420.}	97.5014	0.335985	newly 62 weeks cumul	94 760.2	75 918.	{53 555., 90 607.}	19.8841	0.142416
2	newly 3 weeks spec	2838.	4619.37	{3657., 5683.}	65.2956	0.21644	newly 3 weeks cumul	5925.	8815.6	{7038., 10 946.}	51.5932	0.179721	newly 5 weeks spec	5914.	9175.33	{8114., 10 354.}	58.1713	0.200032	newly 5 weeks cumul	16 050.	25 007.4	{20 418., 29 810.}	58.471	0.199502	newly 58 weeks cumul	82 551.	84 998.6	{76 039., 92 336.}	11.675	0.0548488
3	newly 3 weeks spec	1557.	165.75	{0., 381.}	92.1715	1.93941	newly 3 weeks cumul	2607.	274.94	{1., 594.}	91.842	1.77903	newly 5 weeks spec	6571.	635.93	{0., 1538.}	92.4074	2.22981	newly 5 weeks cumul	12 454.	1246.81	{2., 2849.}	92.3757	2.0065	newly 63 weeks cumul	90 095.	59 628.1	{5., 89 589.}	34.2989	0.85459
4	newly 3 weeks spec	302.	263.15	{0., 256.}	149.725	1.72623	newly 3 weeks cumul	421.	334.75	{1., 375.}	139.969	1.48212	newly 5 weeks spec	3492.	1401.83	{0., 2878.}	109.736	2.38233	newly 5 weeks cumul	5026.	2519.36	{1., 4210.}	118.469	2.02902	newly 67 weeks cumul	90 300.	56 716.2	{2., 92 086.}	37.988	1.30711
5	newly 3 weeks spec	841.	125.07	{0., 266.}	92.1653	1.90853	newly 3 weeks cumul	1316.	203.53	{2., 443.}	91.2378	1.69942	newly 5 weeks spec	3644.	587.29	{0., 1381.}	93.3598	2.27912	newly 5 weeks cumul	6776.	1073.49	{2., 2423.}	92.9151	1.99581	newly 64 weeks cumul	88 461.	56 277.7	{2., 89 878.}	37.4965	1.21886
6	newly 3 weeks spec	1468.	169.68	{0., 632.}	88.951	2.11619	newly 3 weeks cumul	2440.	299.86	{1., 1151.}	88.2328	1.85763	newly 5 weeks spec	5118.	634.26	{0., 2319.}	88.9168	2.40627	newly 5 weeks cumul	10 351.	1275.6	{1., 4784.}	88.7099	2.09275	newly 62 weeks cumul	91 404.	49 508.7	{1., 90 333.}	46.0745	1.39273
7	newly 3 weeks spec	1624.	264.52	{1., 799.}	86.3793	1.71928	newly 3 weeks cumul	2704.	484.47	{4., 1442.}	85.6498	1.54632	newly 5 weeks spec	5875.	781.89	{0., 2436.}	87.4609	2.09165	newly 5 weeks cumul	11 979.	1735.47	{5., 5316.}	86.8068	1.80191	newly 60 weeks cumul	90 884.	55 105.6	{7., 86 392.}	39.6369	0.976632
8	newly 3 weeks spec	1359.	328.17	{0., 854.}	88.9838	1.67656	newly 3 weeks cumul	2461.	560.9	{3., 1372.}	87.744	1.52515	newly 5 weeks spec	5170.	1135.08	{0., 3321.}	87.6395	1.88943	newly 5 weeks cumul	10 407.	2337.44	{5., 6389.}	87.9372	1.6852	newly 62 weeks cumul	92 361.	61 012.7	{7., 89 267.}	34.1006	0.800683
9	newly 3 weeks spec	953.	142.13	{0., 456.}	88.553	1.75949	newly 3 weeks cumul	1486.	252.41	{2., 810.}	87.181	1.54115	newly 5 weeks spec	4055.	509.1	{0., 1671.}	89.8688	2.06584	newly 5 weeks cumul	7477.	1035.38	{2., 3322.}	89.1954	1.81977	newly 62 weeks cumul	92 411.	56 843.7	{3., 90 287.}	38.6551	1.02565
10	newly 3 weeks spec	629.	146.93	{0., 377.}	93.9094	1.80403	newly 3 weeks cumul	952.	208.31	{2., 530.}	92.167	1.59158	newly 5 weeks spec	4393.	1092.92	{0., 3272.}	96.0278	2.22021	newly 5 weeks cumul	6990.	1736.37	{2., 5024.}	95.7538	1.93282	newly 66 weeks cumul	75 317.	65 542.6	{2., 91 027.}	28.8544	0.82142
11	newly 3 weeks spec	2514.	3257.78	{1771., 4466.}	42.6118	0.161896	newly 3 weeks cumul	4845.	5790.4	{3032., 7984.}	34.0784	0.136892	newly 5 weeks spec	6044.	8438.86	{5362., 10 285.}	47.3802	0.172541	newly 5 weeks cumul	15 023.	19 885.6	{11 635., 25 646.}	42.8303	0.1609	newly 60 weeks cumul	84 746.	84 634.2	{76 852., 91 473.}	6.93043	0.0332571
12	newly 3 weeks spec	1011.	109.13	{0., 354.}	89.2057	1.91858	newly 3 weeks cumul	1634.	201.57	{1., 650.}	87.7974	1.68344	newly 5 weeks spec	4508.	348.79	{0., 1198.}	92.2629	2.28106	newly 5 weeks cumul	8400.	751.47	{2., 2607.}	91.0539	2.00561	newly 61 weeks cumul	92 398.	50 533.8	{2., 88 054.}	45.3638	1.27257
13	newly 3 weeks spec	2062.	1973.91	{767., 3352.}	38.0626	0.216685	newly 3 weeks cumul	3828.	3635.61	{1435., 6028.}	36.6627	0.20539	newly 5 weeks spec	4926.	5154.44	{2050., 8330.}	36.9618	0.206772	newly 5 weeks cumul	12 079.	12 129.6	{4828., 19 868.}	37.1149	0.206142	newly 59 weeks cumul	90 371.	79 543.6	{39 550., 93 038.}	13.5261	0.105755
14	newly 3 weeks spec	207.	112.74	{0., 243.}	107.556	1.40732	newly 3 weeks cumul	282.	183.75	{1., 406.}	109.337	1.13167	newly 5 weeks spec	1855.	529.55	{0., 1313.}	98.0577	1.99184	newly 5 weeks cumul	2833.	971.58	{3., 2311.}	99.6548	1.63554	newly 64 weeks cumul	71 218.	55 082.9	{4., 91 123.}	41.7738	1.05447
15	newly 3 weeks spec	874.	129.22	{0., 245.}	92.5561	2.08345	newly 3 weeks cumul	1434.	208.55	{2., 379.}	91.8529	1.79251	newly 5 weeks spec	4922.	619.72	{0., 1349.}	92.1536	2.54088	newly 5 weeks cumul	8480.	1125.02	{2., 2320.}	92.2196	2.15519	newly 64 weeks cumul	79 509.	53 330.2	{3., 90 307.}	40.3332	1.22279
16	newly 3 weeks spec	582.	190.06	{0., 222.}	103.423	1.72565	newly 3 weeks cumul	805.	294.14	{2., 376.}	103.58	1.46862	newly 5 weeks spec	4823.	830.99	{0., 953.}	95.52	2.31401	newly 5 weeks cumul	7337.	1566.25	{2., 1757.}	97.4675	1.96414	newly 64 weeks cumul	91 266.	57 544.3	{3., 91 708.}	37.3526	1.1435
17	newly 3 weeks spec	2320.	2498.89	{1220., 3592.}	34.1409	0.157858	newly 3 weeks cumul	4411.	4560.55	{2588., 6506.}	30.7815	0.146835	newly 5 weeks spec	5419.	6460.41	{2775., 8762.}	38.6974	0.1648	newly 5 weeks cumul	13 371.	15 286.2	{7493., 21 388.}	36.4794	0.159684	newly 59 weeks cumul	87 876.	84 272.3	{74 844., 93 080.}	10.0637	0.0555347
18	newly 3 weeks spec	1347.	335.53	{1., 988.}	79.0965	1.45781	newly 3 weeks cumul	2379.	610.38	{4., 1751.}	78.3531	1.3379	newly 5 weeks spec	4153.	1010.6	{0., 2993.}	80.0356	1.67264	newly 5 weeks cumul	9012.	2217.16	{5., 6485.}	79.6247	1.47132	newly 60 weeks cumul	79 943.	59 461.2	{6., 90 538.}	33.2714	0.790805
19	newly 3 weeks spec	927.	139.2	{0., 330.}	88.4984	1.61094	newly 3 weeks cumul	1494.	246.37	{3., 576.}	87.0194	1.41942	newly 5 weeks spec	4254.	503.92	{0., 1179.}	90.5247	1.97619	newly 5 weeks cumul	7918.	1015.14	{3., 2378.}	89.8608	1.72552	newly 62 weeks cumul	93 229.	59 264.8	{5., 90 683.}	36.4465	0.841097
20	newly 3 weeks spec	53.	619.13	{0., 210.}	1193.08	1.33148	newly 3 weeks cumul	74.	820.96	{1., 301.}	1126.46	1.03618	newly 5 weeks spec	1059.	1832.87	{0., 1670.}	220.037	2.02792	newly 5 weeks cumul	1395.	3871.56	{1., 2662.}	320.459	1.63922	newly 67 weeks cumul	91 568.	53 830.6	{2., 92 645.}	41.7545	1.47297
21	newly 3 weeks spec	845.	129.11	{0., 351.}	90.5645	1.97228	newly 3 weeks cumul	1345.	208.28	{1., 552.}	89.7264	1.79685	newly 5 weeks spec	4282.	595.91	{0., 1816.}	91.6233	2.33078	newly 5 weeks cumul	7677.	1092.46	{1., 3184.}	91.2831	2.08195	newly 64 weeks cumul	83 732.	50 607.6	{1., 90 581.}	43.691	1.48963
22	newly 3 weeks spec	804.	319.29	{0., 667.}	109.066	1.96947	newly 3 weeks cumul	1237.	465.53	{2., 1065.}	105.797	1.68289	newly 5 weeks spec	4322.	1258.52	{0., 3078.}	100.126	2.28979	newly 5 weeks cumul	7480.	2454.22	{3., 5667.}	103.124	1.98684	newly 65 weeks cumul	84 795.	57 359.8	{4., 91 637.}	35.8431	0.988454
23	newly 3 weeks spec	2606.	2534.77	{1537., 3128.}	19.0579	0.0971084	newly 3 weeks cumul	5006.	4798.41	{2949., 5995.}	17.8927	0.0920205	newly 5 weeks spec	5905.	6080.15	{3623., 7321.}	21.8555	0.105593	newly 5 weeks cumul	15 055.	14 985.3	{8846., 18 323.}	19.664	0.0979126	newly 58 weeks cumul	84 036.	82 469.8	{66 019., 92 734.}	10.141	0.0542712
24	newly 3 weeks spec	1803.	261.88	{1., 720.}	85.4753	1.58915	newly 3 weeks cumul	3172.	517.13	{4., 1403.}	83.697	1.42871	newly 5 weeks spec	5220.	680.05	{0., 1995.}	86.9722	1.82231	newly 5 weeks cumul	11 528.	1627.92	{5., 4610.}	85.8786	1.61089	newly 58 weeks cumul	92 404.	53 515.	{8., 85 115.}	42.0858	0.869922
25	newly 3 weeks spec	784.	78.09	{0., 186.}	90.1492	1.68491	newly 3 weeks cumul	1188.	152.21	{2., 365.}	87.9907	1.46872	newly 5 weeks spec	4655.	227.61	{0., 496.}	95.1104	2.2701	newly 5 weeks cumul	7743.	515.21	{3., 1183.}	93.3461	1.90416	newly 61 weeks cumul	89 551.	51 437.2	{4., 87 434.}	42.7674	1.1581
26	newly 3 weeks spec	2337.	2829.85	{1566., 3878.}	36.5841	0.150098	newly 3 weeks cumul	4561.	5183.26	{2895., 7011.}	30.8932	0.132906	newly 5 weeks spec	5505.	7067.17	{4412., 9079.}	39.8992	0.157394	newly 5 weeks cumul	13 758.	17 000.8	{9967., 22 644.}	36.6419	0.14779	newly 59 weeks cumul	89 914.	84 015.4	{76 427., 93 004.}	8.85205	0.047667
27	newly 3 weeks spec	3178.	5318.87	{3779., 6369.}	67.5289	0.215073	newly 3 weeks cumul	6240.	9503.29	{6621., 11 303.}	53.5623	0.17906	newly 5 weeks spec	6900.	11 374.6	{9446., 12 568.}	64.8494	0.214128	newly 5 weeks cumul	18 218.	29 465.8	{22 786., 33 736.}	61.7399	0.203164	newly 60 weeks cumul	90 939.	86 079.6	{76 880., 92 711.}	6.18487	0.0282817
28	newly 3 weeks spec	1642.	702.28	{0., 3091.}	103.423	1.94476	newly 3 weeks cumul	2833.	1054.79	{2., 4559.}	98.4804	1.72796	newly 5 weeks spec	6075.	2569.7	{0., 14 242.}	99.7995	2.1118	newly 5 weeks cumul	12 201.	5188.33	{4., 26 573.}	101.158	1.88687	newly 64 weeks cumul	93 780.	60 206.	{15., 91 211.}	35.8101	0.821185
29	newly 3 weeks spec	2186.	2424.83	{1370., 2920.}	28.2658	0.124901	newly 3 weeks cumul	4260.	4582.67	{2848., 5508.}	25.281	0.113924	newly 5 weeks spec	5160.	5870.16	{3331., 7122.}	29.5678	0.128287	newly 5 weeks cumul	12 858.	14 408.5	{7867., 17 494.}	28.2187	0.12325	newly 58 weeks cumul	82 934.	83 147.3	{73 555., 92 765.}	10.0621	0.0509238
30	newly 3 weeks spec	404.	1383.32	{0., 357.}	407.936	1.86277	newly 3 weeks cumul	607.	1904.36	{1., 473.}	378.926	1.67988	newly 5 weeks spec	3670.	1208.89	{0., 3882.}	99.6984	2.34388	newly 5 weeks cumul	5636.	4481.9	{1., 5538.}	146.267	2.07934	newly 67 weeks cumul	93 251.	59 128.5	{1., 93 474.}	36.7194	1.23192
31	newly 3 weeks spec	169.	2229.29	{0., 436.}	1373.79	1.7527	newly 3 weeks cumul	246.	2971.33	{0., 502.}	1260.63	1.53569	newly 5 weeks spec	2181.	1873.98	{0., 5747.}	149.267	2.26161	newly 5 weeks cumul	3075.	6911.88	{1., 20 081.}	286.887	2.0248	newly 68 weeks cumul	88 759.	55 325.9	{1., 92 231.}	39.4743	1.47708
32	newly 3 weeks spec	874.	360.52	{0., 626.}	109.554	1.85948	newly 3 weeks cumul	1385.	525.15	{1., 961.}	105.835	1.68415	newly 5 weeks spec	4137.	1531.23	{0., 3281.}	104.345	2.2049	newly 5 weeks cumul	7503.	2918.22	{3., 5788.}	106.467	1.95361	newly 65 weeks cumul	77 539.	59 302.8	{3., 92 161.}	35.1359	1.03622
33	newly 3 weeks spec	1347.	315.23	{1., 843.}	89.7424	1.72307	newly 3 weeks cumul	2306.	513.51	{3., 1372.}	88.4818	1.59492	newly 5 weeks spec	4307.	1230.64	{0., 3770.}	90.7783	1.93809	newly 5 weeks cumul	9125.	2412.71	{3., 6925.}	90.2142	1.76414	newly 63 weeks cumul	92 452.	60 271.4	{5., 89 999.}	34.996	0.878333
34	newly 3 weeks spec	2211.	3447.55	{2359., 4807.}	59.825	0.19567	newly 3 weeks cumul	4652.	6550.6	{4705., 9047.}	44.8345	0.153702	newly 5 weeks spec	4361.	7533.89	{4620., 9654.}	76.9573	0.244028	newly 5 weeks cumul	12 123.	19 508.7	{13 345., 26 028.}	64.5996	0.210533	newly 58 weeks cumul	74 850.	82 666.4	{46 368., 91 973.}	21.0187	0.095309
35	newly 3 weeks spec	845.	82.31	{0., 194.}	92.1858	1.88167	newly 3 weeks cumul	1377.	142.8	{2., 355.}	91.4553	1.70797	newly 5 weeks spec	3852.	335.41	{0., 762.}	93.2526	2.18315	newly 5 weeks cumul	7034.	648.66	{3., 1391.}	92.7828	1.9735	newly 63 weeks cumul	93 735.	56 848.9	{4., 90 206.}	39.3579	0.957638
36	newly 3 weeks spec	2415.	3540.	{1897., 4669.}	55.34	0.187486	newly 3 weeks cumul	4659.	6110.97	{3359., 7937.}	42.8631	0.157339	newly 5 weeks spec	5307.	9403.64	{5331., 11 985.}	79.9827	0.243389	newly 5 weeks cumul	13 623.	21 827.9	{13 080., 27 922.}	64.5726	0.207305	newly 61 weeks cumul	83 417.	81 158.8	{37 014., 93 189.}	12.7617	0.0694457
37	newly 3 weeks spec	569.	626.85	{0., 486.}	176.543	1.84297	newly 3 weeks cumul	794.	838.04	{1., 773.}	169.645	1.61559	newly 5 weeks spec	3995.	1647.38	{0., 3812.}	111.548	2.2894	newly 5 weeks cumul	6432.	3710.13	{2., 5774.}	127.026	1.99607	newly 66 weeks cumul	88 408.	59 590.	{2., 93 171.}	34.8431	1.14277
38	newly 3 weeks spec	174.	145.93	{0., 249.}	134.431	1.49246	newly 3 weeks cumul	245.	209.23	{1., 396.}	131.122	1.21963	newly 5 weeks spec	2064.	1040.43	{0., 2163.}	114.9	2.21508	newly 5 weeks cumul	2893.	1685.78	{1., 3432.}	119.426	1.77814	newly 66 weeks cumul	81 546.	53 156.1	{1., 90 889.}	41.1595	1.36063
39	newly 3 weeks spec	1689.	1459.98	{31., 3134.}	53.5394	0.512006	newly 3 weeks cumul	3181.	2601.98	{65., 5449.}	50.6916	0.472877	newly 5 weeks spec	4543.	4173.77	{60., 8342.}	54.0486	0.544169	newly 5 weeks cumul	10 645.	9366.18	{177., 19 393.}	52.729	0.505592	newly 60 weeks cumul	70 294.	77 193.9	{38 367., 92 931.}	26.432	0.237024
40	newly 3 weeks spec	2733.	4221.69	{2338., 6622.}	61.824	0.201908	newly 3 weeks cumul	5234.	7280.45	{4368., 11 373.}	48.4992	0.168527	newly 5 weeks spec	6145.	10 366.8	{5958., 13 815.}	72.2648	0.232804	newly 5 weeks cumul	15 669.	24 978.9	{15 173., 36 068.}	64.2882	0.210458	newly 61 weeks cumul	84 270.	81 449.4	{53 274., 89 118.}	10.8106	0.0572556
41	newly 3 weeks spec	2085.	3639.73	{1519., 5191.}	86.6748	0.297228	newly 3 weeks cumul	3973.	6083.04	{2734., 8896.}	67.998	0.259326	newly 5 weeks spec	5334.	9937.63	{4992., 12 947.}	94.4948	0.316745	newly 5 weeks cumul	12 888.	22 711.8	{11 037., 30 433.}	85.7961	0.296362	newly 62 weeks cumul	72 211.	73 707.6	{53 891., 90 643.}	21.3258	0.0974976
42	newly 3 weeks spec	641.	296.54	{0., 464.}	111.438	1.85705	newly 3 weeks cumul	992.	405.61	{2., 689.}	106.43	1.65896	newly 5 weeks spec	4341.	1411.28	{0., 3697.}	96.9781	2.30759	newly 5 weeks cumul	7191.	2578.43	{2., 6428.}	100.745	2.01399	newly 66 weeks cumul	73 182.	57 124.6	{2., 92 371.}	40.0555	1.1157
43	newly 3 weeks spec	1358.	199.89	{0., 538.}	86.1495	1.70009	newly 3 weeks cumul	2359.	387.09	{3., 1039.}	84.7821	1.48447	newly 5 weeks spec	4809.	548.8	{0., 1494.}	88.5881	2.00826	newly 5 weeks cumul	9757.	1272.21	{4., 3313.}	87.1771	1.72076	newly 59 weeks cumul	92 457.	53 838.3	{4., 85 684.}	41.7694	0.972545
44	newly 3 weeks spec	944.	174.98	{0., 240.}	95.4258	2.06045	newly 3 weeks cumul	1464.	258.56	{2., 404.}	94.0055	1.83023	newly 5 weeks spec	6185.	916.54	{0., 1099.}	94.8682	2.5675	newly 5 weeks cumul	10 162.	1628.6	{2., 1879.}	95.2013	2.20989	newly 65 weeks cumul	91 937.	53 216.6	{3., 91 395.}	42.3223	1.27569
45	newly 3 weeks spec	599.	153.69	{0., 481.}	94.1018	1.87036	newly 3 weeks cumul	910.	246.02	{1., 762.}	93.6132	1.63333	newly 5 weeks spec	4808.	684.39	{0., 2283.}	91.0984	2.42897	newly 5 weeks cumul	7429.	1272.83	{2., 4553.}	91.452	2.08983	newly 64 weeks cumul	82 950.	55 478.1	{2., 91 095.}	37.8308	1.15892
46	newly 3 weeks spec	564.	166.55	{0., 217.}	102.711	1.60655	newly 3 weeks cumul	898.	263.44	{2., 380.}	100.178	1.42915	newly 5 weeks spec	3017.	769.06	{0., 1198.}	102.433	2.03516	newly 5 weeks cumul	5294.	1423.3	{2., 2097.}	102.646	1.75135	newly 64 weeks cumul	85 120.	61 873.5	{5., 91 763.}	30.3003	0.929695
47	newly 3 weeks spec	765.	159.09	{0., 430.}	92.3438	1.98022	newly 3 weeks cumul	1158.	226.25	{1., 587.}	90.7435	1.76844	newly 5 weeks spec	3839.	1163.91	{0., 3341.}	97.6194	2.3458	newly 5 weeks cumul	6738.	1869.66	{1., 5161.}	95.946	2.04949	newly 66 weeks cumul	82 631.	56 738.8	{1., 92 629.}	37.9435	1.30169
48	newly 3 weeks spec	268.	193.5	{0., 269.}	136.866	1.71532	newly 3 weeks cumul	370.	263.69	{1., 369.}	132.516	1.46205	newly 5 weeks spec	2437.	1128.62	{0., 2372.}	117.672	2.2451	newly 5 weeks cumul	3631.	1963.76	{2., 3578.}	123.903	1.92347	newly 66 weeks cumul	91 693.	59 228.8	{2., 92 033.}	35.7126	1.07157
49	newly 3 weeks spec	151.	1530.97	{0., 125.}	1074.18	1.82607	newly 3 weeks cumul	195.	2341.84	{0., 162.}	1257.57	1.5369	newly 5 weeks spec	3032.	1459.48	{0., 692.}	123.868	2.59823	newly 5 weeks cumul	3868.	5533.96	{0., 1564.}	215.321	2.26855	newly 68 weeks cumul	88 820.	52 877.3	{0., 92 007.}	42.0168	1.65702
50	newly 3 weeks spec	2772.	2278.88	{1072., 3581.}	30.7468	0.184445	newly 3 weeks cumul	5085.	4182.99	{2096., 6470.}	30.0946	0.178307	newly 5 weeks spec	6948.	5912.89	{2635., 8610.}	27.4302	0.164439	newly 5 weeks cumul	16 731.	13 985.2	{6800., 21 142.}	28.6757	0.17143	newly 59 weeks cumul	91 561.	83 433.6	{68 095., 93 073.}	9.83549	0.0558099
